# Contrasting nitrogen fertilization treatments impact xylem gene expression and secondary cell wall lignification in *Eucalyptus*

**DOI:** 10.1186/s12870-014-0256-9

**Published:** 2014-09-28

**Authors:** Eduardo Leal Oliveira Camargo, Leandro Costa Nascimento, Marçal Soler, Marcela Mendes Salazar, Jorge Lepikson-Neto, Wesley Leoricy Marques, Ana Alves, Paulo José Pereira Lima Teixeira, Piotr Mieczkowski, Marcelo Falsarella Carazzolle, Yves Martinez, Ana Carolina Deckmann, José Carlos Rodrigues, Jacqueline Grima-Pettenati, Gonçalo Amarante Guimarães Pereira

**Affiliations:** Universidade Estadual de Campinas; UNICAMP; Instituto de Biologia; Departamento de Genética, Evolução e Bioagentes; Laboratório de Genômica e Expressão, Campinas, Brazil; Laboratoire de Recherche en Sciences Végétales, UMR 5546: CNRS - Université de Toulouse III (UPS), Auzeville, BP 42617, F-31326 Castanet-Tolosan, France; Tropical Research Institute of Portugal (IICT), Forestry and Forest Products Group, Tapada da Ajuda, Lisboa, Portugal; Centro de Estudos Florestais, Tapada da Ajuda, Lisboa, Portugal; University of North Carolina at Chapel Hill, Chapel Hill, NC USA; Fédération de Recherche “Agrobiosciences, Interactions et Biodiversité”, 24 Chemin de borde rouge, BP 42617, 31326 Castanet-Tolosan, France

**Keywords:** Eucalyptus, Lignin, Lignocellulosic biomass, Nitrogen fertilization, Wood, RNA sequencing

## Abstract

**Background:**

Nitrogen (N) is a main nutrient required for tree growth and biomass accumulation. In this study, we analyzed the effects of contrasting nitrogen fertilization treatments on the phenotypes of fast growing *Eucalyptus* hybrids (*E. urophylla* x *E. grandis*) with a special focus on xylem secondary cell walls and global gene expression patterns.

**Results:**

Histological observations of the xylem secondary cell walls further confirmed by chemical analyses showed that lignin was reduced by luxuriant fertilization, whereas a consistent lignin deposition was observed in trees grown in N-limiting conditions. Also, the syringyl/guaiacyl (S/G) ratio was significantly lower in luxuriant nitrogen samples. Deep sequencing RNAseq analyses allowed us to identify a high number of differentially expressed genes (1,469) between contrasting N treatments. This number is dramatically higher than those obtained in similar studies performed in poplar but using microarrays. Remarkably, all the genes involved the general phenylpropanoid metabolism and lignin pathway were found to be down-regulated in response to high N availability. These findings further confirmed by RT-qPCR are in agreement with the reduced amount of lignin in xylem secondary cell walls of these plants.

**Conclusions:**

This work enabled us to identify, at the whole genome level, xylem genes differentially regulated by N availability, some of which are involved in the environmental control of xylogenesis. It further illustrates that N fertilization can be used to alter the quantity and quality of lignocellulosic biomass in *Eucalyptus,* offering exciting prospects for the pulp and paper industry and for the use of short coppices plantations to produce second generation biofuels.

**Electronic supplementary material:**

The online version of this article (doi:10.1186/s12870-014-0256-9) contains supplementary material, which is available to authorized users.

## Background

Plants have the capability to produce biomass from carbon dioxide, light energy, water and nutrients, the last two being important limiting factors to growth and development [1]. With the current global demand for energy, plant biomass has become the main gamble as a renewable and environmentally cost-effective new feedstock to chemicals and biofuel production [2-5]. This bioenergy potential now converge the interests to understand the molecular mechanisms that control the plant biomass production and improve plant biomass yield [6].

Plant biomass yield is determined by a number of environmental factors, such as the efficiencies of the capture and conversion of solar energy, water and nutrients. Among nutrients, nitrogen (N) is considered as the main limiting factor for plant growth and development [7] and used to increase the productivity of agricultural crops and commercial forests by the application of large quantities of costly fertilizers.

Despite the well-documented effects of the N fertilizers on plant growth and development rates, the molecular mechanisms by which these responses are converted in phenotypic traits are still not satisfactorily elucidated. More importantly, N fertilizers seem to display effects on wood properties, which have a profound impact on commercial trees used for paper, pulp and biomass, but little is known about the responses underlying these effects [8-14]. This question is important and timely because wood is one of the most important source of terrestrial biomass and a major feedstock for pulp and paper production. Wood is also expected to be increasingly exploited for the production of chemicals and bioenergy within the context of sustainable development [3-5].

Because of their fast growth, valuable wood properties, wide adaptability to soils and climates, and ease of management through coppicing, *Eucalyptus* species and their hybrids are the most widely planted hardwood in the world [15,16]. These world’s leading sources of lignocellulosic biomass supply high quality raw material for pulp, paper, timber and energy [17] and could also provide sustainable and cost-efficient production of lignocellulosic second-generation biofuels [5,18]. However, the main limitation to this objective is wood recalcitrance to degradation, which is dependent on the structure and composition of the lignified secondary cell walls [3-5].

The secondary cell walls (SCW) are mainly composed of cellulose, hemicelluloses and lignin. Lignin is the second most abundant plant biopolymer deposited predominantly in the secondary walls of tracheary elements and fibers in wood [19]. It is produced by the dehydrogenative polymerization of essentially three different hydroxycinnamyl alcohols, the *p*-coumaryl, coniferyl, and sinapyl alcohols, that differ in the degree of methoxylation at the C_3_ and C_5_ positions of the aromatic ring [20]. The deposition of this polymer confers rigidity and also protects the cell wall polysaccharides from pathogens and microbial degradation [21]. This high resistance to degradation is also one of the most important industrial limitations, where lignin impairs the accessibility of cellulose during kraft pulping as well as during saccharification, a key step in the process of bioethanol production. Therefore, there is much interest in understanding the molecular mechanisms underlying xylogenesis and SCW formation in order to tailor plants traits better adapted to industrial purposes.

Increasing evidences support that not only the quantity but also the quality of lignocellulosic material can be substantially altered by manipulating the environment in which feedstock species are grown [22], and ref therein. Among the diverse managements procedures displaying effects on wood quality, manipulating nitrogen availability, one of the most limiting nutrients for tree growth and carbon sequestration, appears as a promising strategy.

In poplar, nitrogen availability has been described to influence growth and development as well as xylogenesis. Fiber morphology, SCW structure and composition were modified in response to high N supply [8,12,13], including lignification pattern [9,11,13]. Further mRNA profiles analysis showed that nitrogen fertilization had overlapping effects with tension wood formation [10] and also pointed some candidate genes potentially related to xylem hydraulic and structural traits [13]. Moreover, using pedigree of pseudo-backcrossed hybrid poplar (*Populus trichocarpa × Populus deltoides*), Novaes et al. [23] have shown that N fertilization significantly increased all growth traits as well as the amount of cellulose and hemicelluloses in the wood whereas a decrease of the lignin content was observed.

Since wood formation, and specially SCW lignification, requires a fine temporal and spatial transcriptional regulation and is highly dependent on the environmental conditions [24], it is important to get further insights on the molecular mechanisms affecting wood formation in *Eucalyptus*, one of the most important forest culture worldwide.

In the present work, we explored the effects of nitrogen availability on the phenotypic and transcriptional responses of the fast-growing *Eucalyptus urophylla* × *E. grandis* hybrids. Using next generation sequencing, we performed a comparative xylem transcriptome profiling analysis between limiting and luxuriant N fertilization treatments that highlighted classes of genes differentially expressed upon nitrogen availability. Together with histological and chemical analysis of xylem cell walls, these results illustrate the phenotypic plasticity of the SCW structure and composition in response to nitrogen management.

## Results and discussion

### Nitrogen availability affects Eucalyptus growth and development

**Figure 1:**
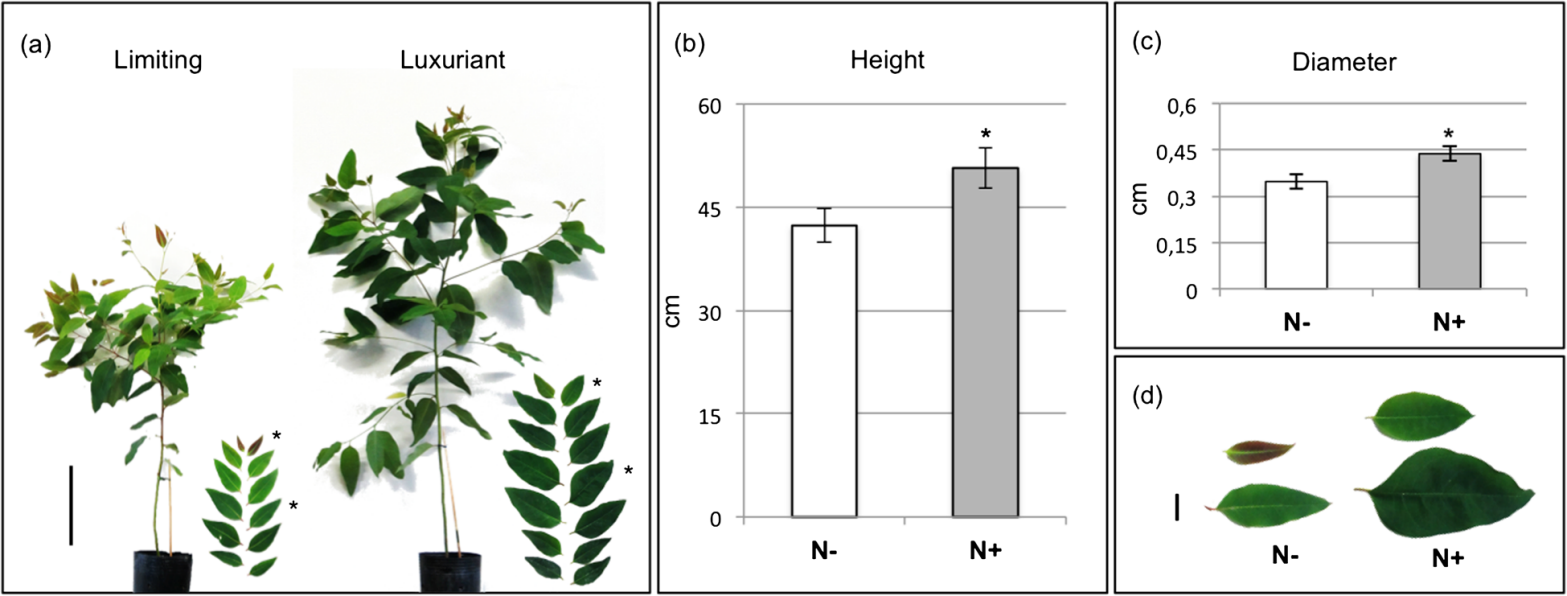
**Effects of limiting and luxuriant N fertilizations on the growth and phenotypes of** ***Eucalyptus*** **hybrids. (a)** Representative young trees and corresponding leaves from the main stem submitted to limiting (N-) and luxuriant (N+) treatments for 30 days. Bar = 20 cm. **(b)** Plant height (cm; Anova: *, *P* < 0.01) **(c)** Plant basal diameter (cm; Anova: *, *P* < 0.01) **(d)** Closer view of the youngest leaves (upper part) and fully expanded leaves (lower part) from plants under N- and N + treatments. Bar = 2 cm.

The effects of nitrogen (N) fertilization were investigated on young trees of *Eucalyptus urophylla* x *E. grandis* (clone IPB2-H15) subjected to four nitrogen (N) treatments, designed as limiting (N-), regular (N) and luxuriant (N + and NO_3_). The availability of N resulted in marked differences in plants’ growth and development. The limiting (N-) and luxuriant (N+) treatments generated the most contrasting phenotypes (Figure 1). On Figure 1a, representative plants grown in N- and N+, respectively are presented. The plants subjected to luxuriant nitrogen fertilization (N+) displayed an increase in plant height (16%) and stem diameter (20%) as compared to plants supplemented with limiting N (N-) (Figure 1b, c). Both luxuriant treatments (N + and NO_3_) similarly contributed to enhance tree growth.

Leaves dimensions and color were also influenced by N availability. Plants grown under N- presented dramatically smaller and lighter green-colored leaves as compared with those from plants grown under regular and especially luxuriant N. The Figure 1d shows a closer-up of representative leaves from the most contrasting treatments (N- and N+). The leaves from N + plants exhibited a dark green color indicating higher chlorophyll content than those from limiting N and even from regular N supply. Interestingly, we observed that the young leaves developed under N limitation presents a purple color, which likely corresponds to anthocyanin accumulation, a common indicator of biotic and abiotic stresses. In *Arabidopsis*, the induction of anthocyanin synthesis in response to N stress was proposed to be an adaptive mechanism controlled by the *NLA* gene [25]. Anthocyanin and flavonoids are products of phenylpropanoid metabolism and, in eucalyptus’ xylem, the supplementation of flavonoids was described to promote alterations at secondary cell walls, especially lignin [26].

### Nitrogen fertilization impacts Eucalyptus secondary cell walls composition and structure

**Figure 2:**
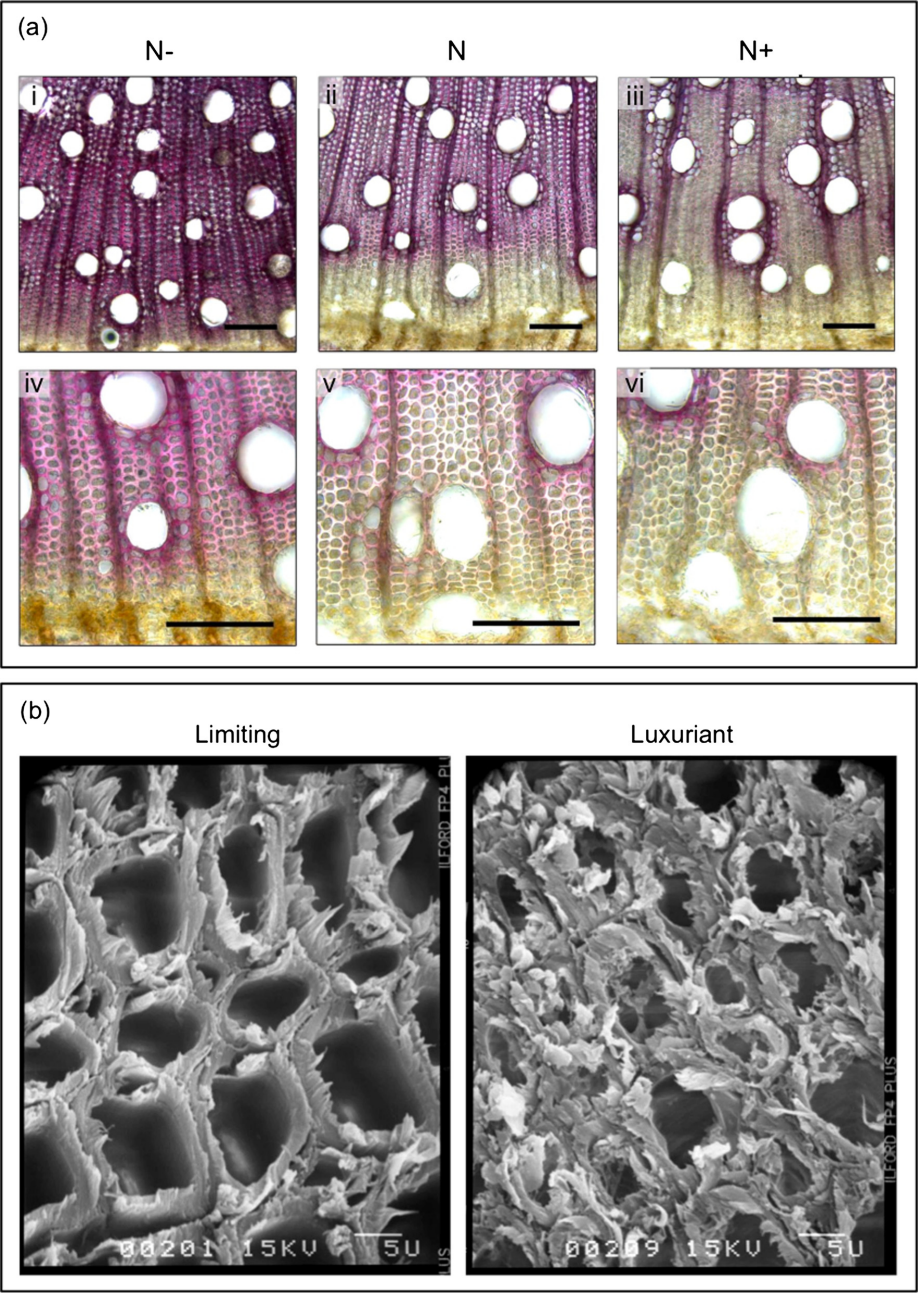
**Effects of N fertilization on** ***Eucalyptus urophylla*** **×** ***E. grandis*** **xylem secondary cell walls. (a)** Phloroglucinol-HCl staining of the basal stem sections from samples grown under N fertilization treatments (N-, N and N+). Bar = 100 μm. **(b)** SEM images from xylem secondary cell walls under limiting (N-) and luxuriant (N+) treatments.

The lignification patterns of xylem SCW of *Eucalyptus* trees were evaluated by staining sections from the basal part of the main stems with phloroglucinol-HCl (Figure 2a). The intensity of the staining with phloroglucinol is usually considered as being proportional to the lignin content. As evidenced by the strong red staining, the xylem cell walls of plants grown in limiting nitrogen conditions (N-) appeared to contain more lignin (Figure 2a-i) than those of trees supplied with regular N (N) (Figure 2a-ii). An opposite and clear trend was observed in the xylem cell walls of N + trees (Figure 2a-iii): the parenchyma fibers showed a lower degree of lignification as indicated by a very faint staining, whereas the red coloration was mostly concentrated in cells surrounding the vessels. In agreement with our observations, high nitrogen fertilization was also described to decrease lignification deposition pattern in poplar’s xylem [9,11,12].

In addition, similar patterns of lignification were observed with both luxuriant (N+) and (NO3-) treatments (data not shown). It seems therefore that, in *Eucalyptus*, the quantity instead of the source of N supplied promotes the main differences in the lignin content of xylem SCW. Closer observations of xylem cells located close to the cambial zone revealed a fainter staining in N + plants especially as compared to N- (Figure 2a-iv, v, vi), suggesting that an excess of N also delays lignin deposition.

The SCW of *Eucalyptus* xylem were further analyzed by scanning electron microscopy (Figure 2b). The comparison between N + and N- treated plants revealed dramatic differences: N + treated plants seemed to present much weaker cell walls, as evidenced by their squashed shape, in contrast to N- treated plants, which presents cells with a regular shape, apparently almost intact. These results strongly suggest profound alterations of the mechanical properties of the cell walls of plants grown under an excess of N, probably as consequence of a reduction of the lignin content. In transgenic poplar, the reduction of lignin affected wood strength and stiffness and also increased proportion of tension wood [27].

The stems harboring these weaker cell walls are likely more flexible and could thus be more susceptible to tension wood formation. Indeed, in some N + samples, we noticed the presence of gelatinous layers of cellulose, also called G-layers, in the lumen of fibers. The G-layers are formed in tension wood in response to mechanical or gravitational stimuli [28-31]. In *Populus nigra,* high N fertilization promoted a two-fold increase on tension wood [32]. Moreover, in the hybrid *Populus trichocarpa* × *P. Deltoides* the G-like layers were shown to be a direct consequence of an excess of N since they were distributed all over the xylem and not restricted to one side of the stem as it is the case for tension wood [8]. In our samples, the G-like layers were found only on one side of the stems, indicating that the formation of tension wood is more probably a stress response to external stimuli and/or to physical constraints, than a direct consequence of a high N supply.

The histological observations were further confirmed by chemical analyses of the xylem cell walls of limiting and luxuriant N fertilized samples. The lignin amounts (total and Klason) were estimated using near-infrared (NIR) spectroscopy (Table 1). Both the total amounts of lignin, as well as the Klason lignin, were significantly reduced in xylem samples of trees grown under luxuriant nitrogen fertilization (N+) as compared to those grown under nitrogen limiting conditions (N-).

**Table 1:** **Lignin chemical analysis from limiting (N-) and luxuriant (N+) treated plants**

	**NIR**				**Pyrolysis**					
	**Total lignin**		**Klason lignin**		**N-**			**N+**		
	**N-**	**N+**	**N-**	**N+**	**S (%)**	**G (%)**	**S/G**	**S (%)**	**G (%)**	**S/G**
R1	27.6	26.7	20.4	19.2	62.7	37.3	1.69	61.4	38.6	1.59
R2	28.0	26.4	21.0	18.8	62.3	37.7	1.65	61.7	38.3	1.61
R3	27.5	26.3	19.9	19.0	62.8	37.2	1.69	61.5	38.5	1.59
Average	27.7	26.5**	20.4	19.0*	62.6	37.4	1.68	61.5	38.4	1.60**
STD	0.28	0.21	0.58	0.19	0.26	0.26	0.02	0.15	0.15	0.01

Lignin content (total and klason) was determined by NIR-PLSR models. Lignin-derived S and G monomers and ratio were calculated by pyrolysis analysis. Each repetition corresponded to 20-polled plants. Percentage was calculated by 20 mg of lignin/ 100 mg dry mass. Anova statistic test: *, *P* < 0.01; **P < 0.001.

In addition, the pyrolysis analysis revealed that the monomeric composition of lignin was also affected by the nitrogen status: the syringyl/guaiacyl (S/G) ratio was significantly lower in luxuriant nitrogen samples (1.60) as compared to low nitrogen plants (1.68). This decreased S/G ratio was the consequence of both a lower quantity of S units and a higher proportion of G units.

### Luxuriant and limiting nitrogen fertilization promote contrasting tendency of xylem gene expression

In order to get an insight in the transcriptomic changes underlying the phenotypic differences induced by the distinct N fertilization treatments, the xylem tissues of 20 young trees of *E. urophylla* × *E. grandis* were sampled and pooled for each N treatment (N-, N, N + and NO_3_). Four libraries were constructed from the total RNA and submitted to Illumina sequencing. The data set construction and the *de novo* assembly (detailed in Additional file 1: Figure S1) led to a large sequence set consisting of 36,781 unigenes.

The reads of each of the four libraries (N-, N, N + and NO_3_) were mapped onto the 36,781 unigenes and the RPKM values (reads per kilobase of exons per million of fragments mapped) were calculated for all the unigenes present in the libraries. Unigenes were considered expressed when the corresponding RPKM ≥ 1 in at least one of the libraries, resulting in a transcriptome composed by 34,919 unigenes (≅95% of the original number of unigenes) (Additional file 2: Table S1).

In order to obtain an accurate picture of the differentially expressed genes (DEG) in response to nitrogen availability, we performed pairwise comparisons between each of the contrasting treatments (N-, N + and NO_3_) against the regular one (N). In addition, a pairwise comparison was performed between each of the two luxuriant (N + and NO_3_) against the limiting (N-) treatment. Genes were considered differently expressed when the fold-change ≥ ±1.5 and the *P*-value ≤0.01. These values were selected according to the total distribution of fold change (Additional file 3: Figure S2) and were identical to those used in previous works approaching N fertilization effects on xylem gene expression [10,13].

The comparison between the most contrasting N treatments (N- and N+), showed very clear differences, with 1,469 differently expressed genes (DEG) exhibiting consistent nitrogen-dependent expression patterns (Additional file 4: Table S2). Nineteen genes exhibiting different expression patterns were validated by qRT PCR (Additional file 5: Table S4) and 10 house keeping genes [33] were verified to be stably expressed between the different treatments. When compared to previous studies using microarray technology [10,13], the RNA deep sequencing dramatically increased (3.8 times more) the number of DEG between two contrasting N treatments.

We compared the distribution of the RPKM of the 34,919 total unigenes to the 1,469 DEGs between N- and N + treatments and classified them into three groups depending on the transcript abundance in xylem: genes with low (RPKM < 25), intermediate (300 > RPKM ≥ 25) or high (RPKM ≥ 300) expression.

**Figure 3:**
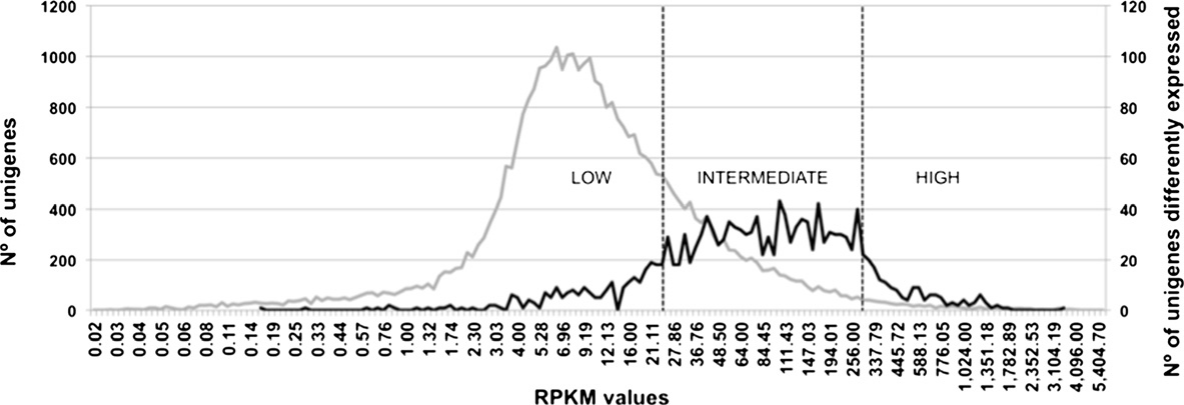
**Distribution of all unigenes and differently expressed genes (DEG) in classes of transcript abundance.** The classes were defined as high (RPKM ≥ 300), intermediate (300 > RPKM ≥ 25) and low (RPKM < 25) RPKM values. Axis “x” in Log_2_ scale. All unigenes in grey, DEG in black.

The distribution of the DEG was dramatically different from that of the total unigenes (Figure 3). While only a small proportion of the DEG were present in the low-expressed class, the vast majority of the total unigenes were expressed at low levels with a large peak between 5–9 RPKM values. Most of the DEG was found in the intermediate-class, which exhibited a plateau-like shape ranging between 27 and 300 RPKM. The number of DEG present in the high-expressed class was low but in significantly higher proportion as compared to the proportion of total unigenes present in this category. These results suggest that the main differences occurring at the xylem phenotype level are likely involving genes with intermediate to high abundance of transcripts.

**Figure 4:**
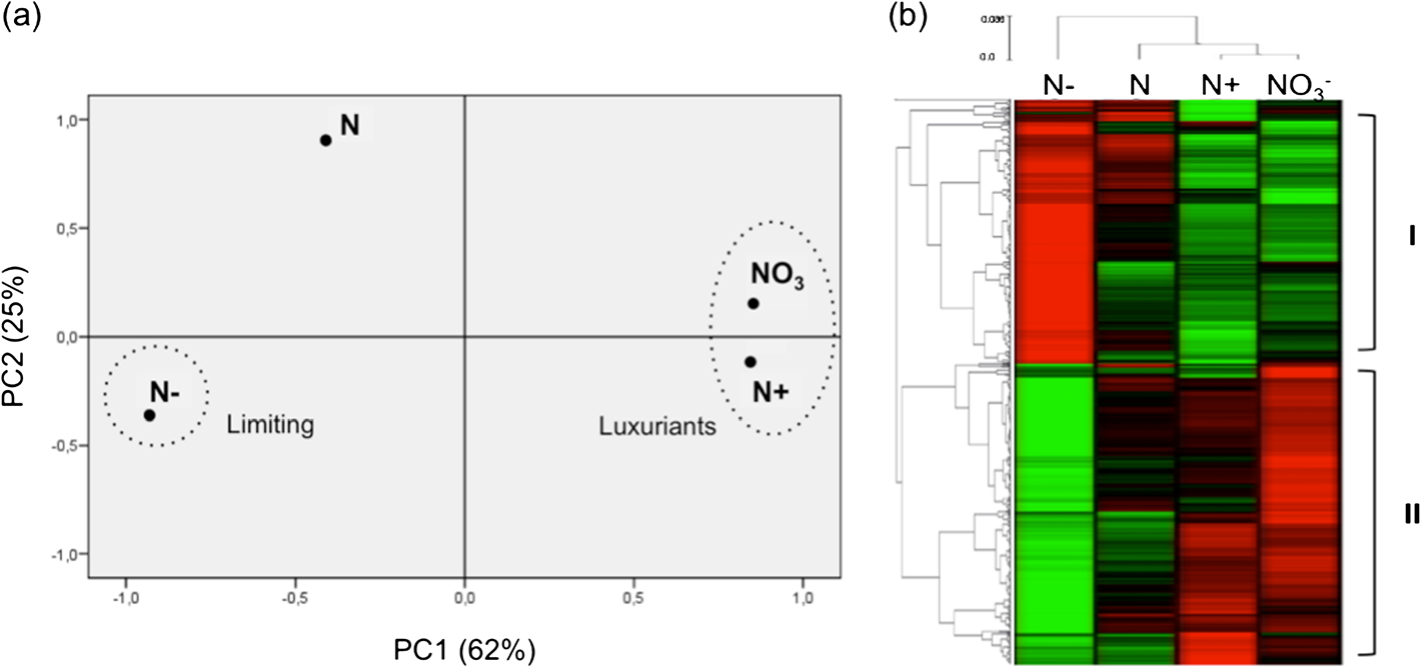
**Global analysis of the differently expressed genes between the contrasting N treatments. (a)** PCA analysis of the DEG in each of the four treatment **(b)** Hierarchical clustering analysis of the DEG showing upregulated genes (red) and downregulated genes (green). (I) genes induced by nitrogen deprivation and repressed by nitrogen luxuriant supply, (II) genes repressed by nitrogen deprivation and induced by nitrogen luxuriant fertilization. All analyses were based on RPKM values for the 4 N treatments and p-value ≤ 0,01.

Standardized log2 RPKM of the DEG for each of the four N treatments were subjected to principal component analysis (PCA), enabling a graphical representation of the correlation between variables (*i.e.* DEG in each treatment) (Figure 4a). The two luxuriant nitrogen treatments (N+, NO3) were grouped together and exhibited a strong positive correlation with the first component that explains 62% of the variance of the data. In sharp contrast, the N limiting treatment (N-) showed a strong negative correlation with the first component. In other words, most of the DEG that were highly expressed in the N-luxuriant treatments were weakly expressed in N-limiting conditions and *vice versa*, indicating two main behaviors of genes according to N availability. These three treatments were weakly correlated to the second component, which explains 25% of the variance, whereas the regular nitrogen treatment (N) was strongly positively correlated to it.

We then performed a hierarchical clustering analysis (HCA, Figure 4b) using the log2 standardized RPKM values of the DEG, which revealed two main trends: some genes (I) were induced by nitrogen deprivation and repressed by nitrogen luxuriant supply, while others (II) showed the opposite trend, being repressed by nitrogen deprivation and induced by nitrogen luxuriant fertilization. These results are in line with the PCA, but also illustrate that even if DEG in both luxuriant treatments exhibit the same tendency, they also display slight different response intensities. A subset of the DEG representing both expression trends (groups I and II) is listed in Table 2 (for a complete list see Additional file 6: Table S3).

**Table 2:** **Subset of the most representative differently expressed genes**

**Gene ID (EucDS)**	**Gene ID (TAIR)**	**Description**	**N+/N-**	
			**Fold**	**P-value**
Nitrogen/Amino acids metabolism				
CD668569.1	AT4G13930.1	Serine hydroxymethyltransferase 4	−1,86	6,3E-11
contig_23319	AT4G13930.1	Serine hydroxymethyltransferase 4	−1,61	3,9E-03
Contig7054	AT4G13930.1	Serine hydroxymethyltransferase 4	−1,85	6,9E-03
de_novo_15907	AT4G13930.1	Serine hydroxymethyltransferase 4	−1,70	1,0E-16
contig_8461	AT2G27820.1	Prephenate dehydratase 1/ Arogenate dehydratase3	−2,26	1,5E-09
contig_15070	AT2G27820.1	Prephenate dehydratase 1/ Arogenate dehydratase3	−2,46	3,3E-03
contig_10858	AT1G08250.1	Arogenate dehydratase 6	−2,19	4,2E-06
de_novo_11386	AT1G08250.1	Arogenate dehydratase 6	−2,71	3,5E-05
de_novo_11507	AT3G44720.1	Arogenate dehydratase 4	−2,33	8,9E-51
contig_10937	AT5G18170.1	Glutamate dehydrogenase 1	−4,14	2,4E-03
de_novo_11901	AT1G12940.1	Nitrate transporter 2.5	−6,57	3,2E-12
de_novo_12308	AT4G13940.4	S-adenosyl-L-homocysteine hydrolase	−1,70	6,7E-03
de_novo_11480	AT1G32450.1	Nitrate transporter 1.5	2,32	2,9E-04
de_novo_11781	AT1G32450.1	Nitrate transporter 1.5	2,25	5,5E-03
contig_94502	AT1G37130.1	Nitrate reductase 2	3,63	6,3E-03
de_novo_11752	AT1G37130.1	Nitrate reductase 2	3,30	3,7E-24
CU395994	AT2G15620.1	Nitrite reductase 1	7,79	7,6E-19
de_novo_11688	AT2G15620.1	Nitrite reductase 1	7,57	8,4E-03
CD670065	AT2G15620.1	Nitrite reductase 1	6,81	1,4E-04
CD669078	AT2G15620.1	Nitrite reductase 1	6,70	4,3E-03
de_novo_11644	AT2G15620.1	Nitrite reductase 1	6,65	1,7E-09
CU396904	AT2G15620.1	Nitrite reductase 1	6,53	6,6E-02
de_novo_19746	AT1G77760.1	Nitrate reductase 1	4,46	1,2E-08
de_novo_11746	AT5G40850.1	Urophorphyrin methylase III	11,16	6,4E-07
CD668172	AT5G35630.3	Glutamine synthetase 2	2,53	7,5E-10
contig_20989	AT5G35630.3	Glutamine synthetase 2	2,43	2,7E-04
de_novo_11573	AT5G57685.1	Glutamine dumper 3	4,38	8,1E-02
de_novo_11536	AT5G57685.1	Glutamine dumper 3	1,84	1,8E-05
Phenylpropanoid metabolism/Lignin Biosynthesis				
contig_10482	AT3G53260.1	Phenylalanine ammonia-lyase 2	−1,56	1,4E-02
contig_21173	AT3G53260.1	Phenylalanine ammonia-lyase 2	−1,68	2,4E-04
contig_16352	AT3G53260.1	Phenylalanine ammonia-lyase 2	−1,65	1,8E-01
contig_3622	AT2G30490.1	Cinnamate-4-hydroxylase	−1,61	3,7E-02
Contig7339	AT2G30490.1	Cinnamate-4-hydroxylase	−1,70	2,1E-02
BD224437.1	AT2G30490.1	Cinnamate-4-hydroxylase	−1,76	8,1E-04
contig_6919	AT1G51680.1	4-coumarate:CoA ligase 1	−1,65	3,4E-02
contig_390	AT4G36220.1	Ferulic acid 5-hydroxylase 1	−1,56	7,5E-02
contig_72476	AT2G40890.1	Cytochrome P450, C3H	−3,48	8,3E-03
de_novo_24790	AT5G54160.1	Caffeic O-methyltransferase1	−1,53	9,5E-32
contig_3127	AT5G60020.1	Laccase 17	−1,56	5,6E-04
contig_23816	AT5G60020.1	Laccase 17	−1,50	1,7E-03
Transcription factors				
contig_3124	AT4G22680.1	Myb domain protein 85	−1,57	4,6E-03
BD377759.1	AT2G23290.1	Myb domain protein 70	−2,14	8,5E-03
contig_18752	AT2G27580.2	A20/AN1-like zinc finger family protein	−2,14	7,4E-02
contig_41808	AT2G27580.2	A20/AN1-like zinc finger family protein	−1,53	6,2E-03
Contig3627	AT3G49930.1	C2H2 and C2HC zinc fingers superfamily protein	−2,08	8,9E-04
contig_6574	AT3G54810.1	Plant-specific GATA-type zinc finger transcription factor family protein	−1,78	1,6E-126
contig_8449	AT1G14920.1	GRAS family transcription factor family protein	−1,98	9,4E-07
contig_3905	AT1G14920.1	GRAS family transcription factor family protein	−1,53	5,7E-03
contig_4005	AT1G78070.1	Transducin/WD40 repeat-like superfamily protein	−1,91	6,5E-03
contig_17263	AT1G24530.1	Transducin/WD40 repeat-like superfamily protein	−1,68	6,0E-02
de_novo_11342	AT3G20640.1	Basic helix-loop-helix (bHLH) DNA-binding superfamily protein	−1,82	7,3E-03
contig_7306	AT1G32640.1	Basic helix-loop-helix (bHLH) DNA-binding family protein	−1,54	7,5E-02
contig_16146	AT2G44745.1	WRKY family transcription factor	−1,55	8,3E-04
contig_4484	AT1G10200.1	GATA type zinc finger transcription factor family protein	2,41	4,9E-03
contig_89946	AT1G80840.1	WRKY DNA-binding protein 40	1,65	4,1E-02
CU401821	AT1G80840.1	WRKY DNA-binding protein 40	2,38	4,9E-11
contig_64900	AT2G38470.1	WRKY DNA-binding protein 33	1,79	1,5E-01
de_novo_11651	AT3G02380.1	CONSTANS-like 2	2,10	2,8E-03
de_novo_12111	AT3G02380.1	CONSTANS-like 2	1,79	1,4E-09
CD668471.1	AT3G02380.1	CONSTANS-like 2	2,00	7,2E-12
de_novo_11383	AT3G49940.1	LOB domain-containing protein 38	6,75	2,8E-43
contig_74960	AT4G31800.2	WRKY DNA-binding protein 18	1,73	1,4E-02
contig_74953	AT5G08790.1	NAC domain transcriptional regulator superfamily protein, ATAF2	1,95	1,7E-06
de_novo_11667	AT5G39660.2	Cycling DOF factor 2	1,94	3,5E-05
contig_12125	AT5G39660.2	Cycling DOF factor 2	1,71	1,7E-03
Other processes				
de_novo_11604	AT2G16060.1	Hemoglobin 1	87,88	1,9E-01
CU401963	AT5G14780.1	Formate dehydrogenase	99,23	6,5E-15
Contig5452	AT5G14780.1	Formate dehydrogenase	69,36	1,7E-05
contig_92267	AT5G14780.1	Formate dehydrogenase	57,00	1,1E-06
contig_94429	AT5G14780.1	Formate dehydrogenase	42,23	4,5E-02
CU396775	AT5G14780.1	Formate dehydrogenase	27,80	3,8E-03

**Figure 5:**
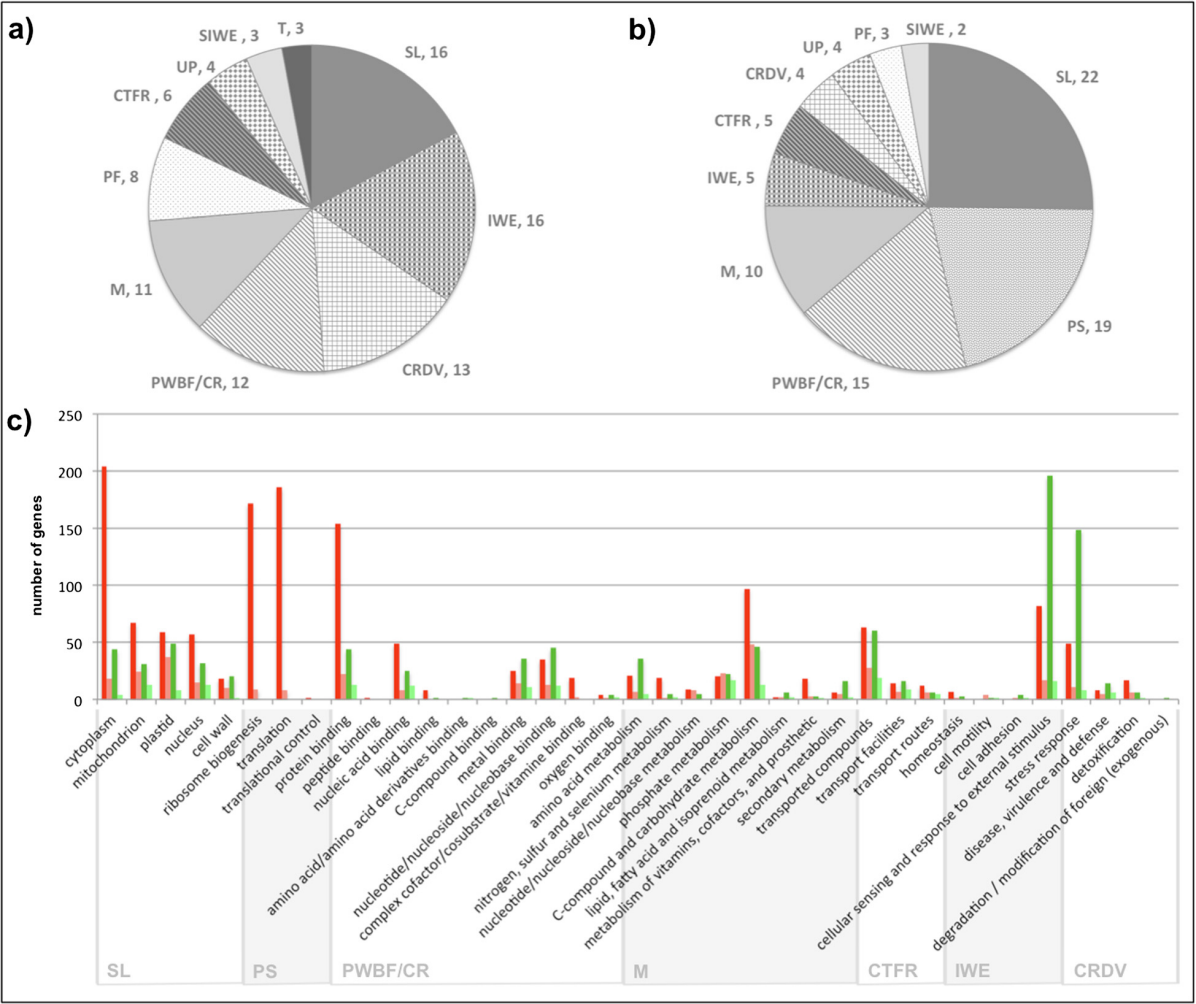
**Functional analysis of the differently expressed genes between the contrasting N treatments. (a)** Top ten categories of the genes induced by nitrogen deprivation and repressed by nitrogen luxuriant supply. **(b)** Top ten categories of the genes repressed by nitrogen deprivation and induced by nitrogen luxuriant fertilization. **(c)** Number of genes differently expressed in the five most represented categories of *Eucalyptus* DEG. Genes induced by nitrogen deprivation and repressed by nitrogen luxuriant supply are presented in dark green for *Eucalyptus* and light green for *Populus* [13]*.* Genes repressed by nitrogen deprivation and induced by nitrogen luxuriant fertilization in dark red for *Eucalyptus* and light red for *Populus*. FUNCAT software [35] categories: SL, subcellular localization; PS, protein synthesis; PWBF/CR, protein with binding function or cofactor requirement; M, metabolism; CTFR, cellular transport, transport facilitation and transport routes; IWE, interaction with the environment; CRDV, cell rescue, defense and virulence; UP, unclassified proteins; PF, protein fate; SIWE, systemic interaction with the environment; T, transcription. **(a)** and **(b)** values in %.

For both group of genes (I and II), the Gene Ontology analysis (Additional file 7: Figure S3) showed that the categories found were similar to those reported in the xylem transcription profiles of three eucalyptus species [34]. FUNCAT analysis (Figure 5), revealed some remarkable differences between group I and II (Figure 5a,b). In group I (genes induced by N deprivation and repressed by N luxuriant fertilization), the categories “interaction with the environment” (IWE), “cell rescue, defense and virulence” (CRDV) and “protein fate” (PF) were enriched when compared to group II. These genes are related to the cellular sensing and response to external stimuli, stress response and protein folding, stabilization, modification and degradation; certainly in response to the stress promoted by N deprivation (described below). In group II (genes repressed by N deprivation and induced by N luxuriant fertilization), the “protein synthesis” category including ribosome biogenesis and protein translation was the second most abundant with 19% of DEG.

The top five most represented categories of each group were also compared with the DEG described in poplar by Plavcová et al. [13]. The number of DEG present in each category was higher when compared to those described in poplar, probably due to the powerfulness of the RNA sequencing technology as compared to microarrays, but also because here we compared two contrasting nitrogen treatments (depletion versus luxuriant) (Figure 5c), while the previous studies use a regular concentration against a high N treatment. In response to nitrogen depletion, the Eucalyptus DEG were more enriched with genes related to cellular sensing and response to external stimuli and stresses, whereas in response to luxuriant N supply, we found a much higher proportion of genes related to protein synthesis. Most of the genes from these categories were not expressed at high levels. This might explain why they were not detected in Plavcová’s study given the lower sensitivity of microrarrays.

### Genes induced by N deprivation and repressed by N luxuriant fertilization

This category was composed of 700 genes, from which 440 showed similarity with *Arabidopsis thaliana* genes and 260 had no hits in the TAIR (The Arabidopsis Information Resource) database. Heat shock and stress-related proteins were the most abundant classes of genes induced by N deprivation and repressed by N luxuriant fertilization (69 proteins, 15.7% from the total of DEG present in this category). *Heat shock proteins* are known to induce cellular responses to environmental stresses and to act as chaperones to protect the correct folding of proteins [36]. The high proportion of these proteins is in line with the stress phenotypes observed on plants grown in N limiting conditions. In creeping grass (*Agrostis stolonifera*) cultivated in N deficient conditions [37], the transcript levels of *small HSP* were up-regulated in immature leaves as compared to mature leaves, suggesting that their abundance play a protective role against protein degradation and aggregation during premature senescence process. Similarly, the increased abundance of *HSP* in Eucalyptus xylem could mitigate the stress response to N deficiency in this highly specialized tissue.

Concerning the SCW polymers, we observed a slight increased of expression of some members of cellulose synthase genes. The genes related to hemicelluloses didn’t show any expression difference except the two transcripts encoding the glycosyltransferase PARVUS involved in xylan synthesis, which were up-regulated by N limiting treatment [38,39] (Additional file 8: Table S5). In agreement with the cytological observations and the chemical analyses, the majority of the genes involved in the lignin biosynthesis pathway were up-regulated in N limiting conditions (N-). They included the genes belonging to the general phenylpropanoid metabolism, encoding *Phenylalanine ammonia lyase* (*PAL*), *Cinnamate-4-hydroxylase* (*C4H*) and *4-Coumarate:CoA ligase* (*4CL*), encoded by 3, 4 and 1 unigenes, respectively, which exhibited significant and coordinated up-regulated expression patterns (Table 2). In poplar, an opposite trend was observed for *PAL1,* which was up-regulated by high N [10] whereas laccases were the only lignin-related genes reported to be induced by nitrogen deprivation [13].

Within the lignin pathway, the transcript encoding *Ferulate 5-hydroxylase* (*F5H*), that catalyzes a key step leading to the formation of the syringyl (S) units in lignin, was also up-regulated in N- treated plants. This corroborates our chemical analyses results, showing an increase of the syringyl units in N limiting conditions as compared to the luxuriant ones (Table 1), and are also reminiscent of what was reported in poplar [9]. In addition, two genes encoding potential orthologs of *Laccase 17* and *Laccase 4,* both specific isoforms demonstrated to be involved in the lignin polymerization step in *Arabidopsis* [40] were up-regulated in N limiting conditions.

**Figure 6:**
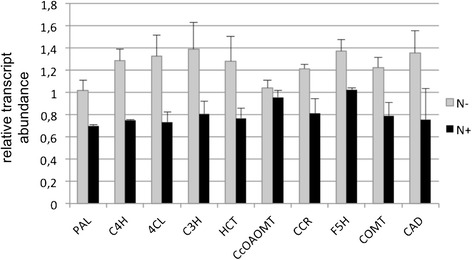
**Expression of selected lignin biosynthesis genes by RT-qPCR.**

Since p-coumarate 3-hydroxylase (C3H), *caffeate/5-hydroxyferulate O-methyltransferase* (*COMT*), *caffeoyl-CoA O-methyltransferase* (*CCoAOMT*), *hydroxycinnamoyl-CoA reductase* (*CCR*), *cinnamyl alcohol dehydrogenase* (*CAD*), were showing the same tendency (P-value ≤ 0.01), but with a fold change below the 1.5 fold cut-off (Additional file 2: Table S1), we decided to further assay by RT-qPCR the transcript levels of all the genes of the lignin biosynthetic pathway (Figure 6 and detailed on Additional file 5: Table S4). The RT-qPCR data further confirmed that all these genes exhibited a significant differential expression between the two most contrasting treatments, confirming the differences highlighted by RNA-seq.

In line with the up-regulation of the genes of the lignin pathway, genes of the upstream shikimate pathway, such as those encoding *Arogenate Dehydratase* (*ADT*) isoforms, were induced in response to N deficiency (Table 2). Since *ADT* enzyme is able to modulate the carbon flux into lignin biosynthesis [41], it is possible that this induction reflects a reorganization of the anabolic metabolism in response to N deficiency. Canton et al. [42] proposed that in xylem the nitrogen released in the form of NH4+ during phenylalanine deamination by *PAL* is strictly destined to synthesize arogenate, which, in turn, is used as substrate for phenylalanine regeneration by *ADT*, instead of making it available to the general protein biosynthesis. Moreover, according to these authors, the phenylpropanoid-nitrogen cycle is a tightly compartmentalized process, separated from the general N metabolism in actively lignifying cells, so that these cells can maintain high rates of lignification without causing a collapse of N content in the plant [42]. Our results showing an increased transcript levels of genes of both the shikimate and the lignin pathways support this hypothesis even under N-limiting conditions.

Besides the up-regulation of the lignin pathway genes, one gene encoding a *Glutamate dehydrogenase* (*GDH*) was also induced in response to N deficiency. GDH is able to catalyse both the amination of 2-oxoglutarate and the deamination of glutamate. Recently, it was suggested that deamination was the predominant role for all the isoenzymes of GDH [43]. In line with these findings, our results suggest that under N deficiency the GDH catalyses glutamate deamination to provide an additional amount of ammonium to the N-deprived tissues. Moreover, a significant up-regulation of genes encoding *Serine Hydroxymethyltransferase 4* (*SHMT*), belonging to the C1 metabolism, was observed in N-limiting conditions. The C1 metabolism, which is especially active in tissues producing methylated compounds such as lignin, has been proposed to be closely connected to lignin biosynthesis through *COMT* and *CCoAOMT* [42,44,45]. In this context, our results suggest that the higher rate of lignin synthesis under nitrogen deficiency is apparently connected to an increase in methyl donor recycling by C1 metabolism: S-adenosyl methionine must be regenerated from S-adenosylhomocysteine associated with 5,6,7,8-tetrahydrofolate, and may rely on formate or on serine and/or glycine as a one carbon donor. In the case of N depletion, serine rather than formate seems to be used as a carbon donor, as suggested by the increased levels of SHMT under these conditions. In addition, the NH4+ released by the glycine to serine recycling could be re-assimilated to produce phenylalanine, as suggested by Cantón et al. [42].

As could be expected from the fact that the SCW formation is mainly regulated at the transcriptional level [24,46-48], many SCW-related transcription factors (TF) were up-regulated in response to N deprivation. They included members of *MYB*, *bHLH*, *WRKY* and *WD40* TF families, which contain members described to play a role in secondary wall formation [45,49-52]. Among these genes, it is worth mentioning that a potential ortholog of the MYB transcription factor, At*MYB85*, described as specifically controlling the biosynthesis of lignin [53], was also up-regulated in N limiting conditions.

Taken together, these results strongly suggest that nitrogen deficiency activates genes associated, directly or indirectly, to lignin synthesis in *Eucalyptus*. These results are in agreement with the histological and chemical analysis, in which lignin levels were significantly higher in xylem from plants grown under limiting N conditions in comparison to those grown under luxuriant N regime.

### Genes repressed by N deprivation and induced by N luxuriant fertilization

A total of 769 genes were present in this category, from which 614 showed sequence similarity with *Arabidopsis thaliana* genes and 155 had no hits in the TAIR database. Genes corresponding to ribosomal proteins were by far the most represented functional category in the group of genes repressed by N deprivation and induced by N luxuriant fertilization (181 genes; 29,5% from the total of DEG identified in this category). Ribosomal proteins are directly correlated with protein synthesis, the principal sink for the nitrogen incorporated by plants.

Several other genes related to amino acids metabolism and transport were also induced by high N fertilization. Among them were genes encoding the *Glutamine dumper 3*, an amino acid exporter highly expressed in *Arabidopsis* vasculature [54] and recently shown to be induced by high N supply in poplar [13].

One of the most up-regulated genes by both luxuriant N conditions encodes a *Formate Dehydrogenase* (*FDH*), which is represented by five unigenes, all very poorly expressed in N- or N conditions. This enzyme is part of the C1 metabolism and known to be induced by several stresses [55]; however, its induction by high nitrogen supply has not been reported before. The oxidation of formate into NADH and CO_2_ by *FDH* may constitute a possible control mechanism to regulate the supply of intermediates to C1 metabolism. So, again, nitrogen availability appears to require adjustments of anabolic metabolism in order to alter the flux of carbon from one pathway to another.

We also identified genes directly correlated to nitrogen uptake in the form of nitrate, such as two potential orthologs of the *Arabidopsis NRT1.5* (At1g32450.1) [56], a low-affinity nitrate transporter. Genes involved in nitrate reduction were represented by one *Nitrate reductase 1* (*NR1*), two *Nitrate reductase 2* (*NR2*) and six *Nitrite reductase* (*NiR*) genes. All showed similar expression patterns, being up-regulated under high nitrogen availability. An *Uroporphyrin III methyltransferase* gene (*UPM1*), which catalyses the biosynthesis of an essential cofactor for *NiR* [57], had a similar trend. Finally, two genes encoding *Glutamine synthetase* (GS), an enzyme involved in ammonium assimilation and re-assimilation processes, were up-regulated in both nitrogen luxuriant treatments. Taken together, these results indicate that under both luxuriant N conditions (N + and NO3+), nitrogen uptake and assimilation by cells are essentially activated

In this context, it is worth noting the strong up-regulation of the *Hemoglobin1 (HB)* gene in both N luxuriant treatments. The expression of *HB* has been already shown to be correlated to the expression profile of a nitrate reductase in maize roots [58]. In addition, several non-symbiotic hemoglobins are known to scavenge nitric oxide (NO) through the production of *S*-nitrosohemoglobin [59]. Thus it is possible that the induction of *HB* under high N supply has a role in NO detoxification to avoid a sustained response associated to NO accumulation as by-product of nitrogen metabolism.

Many transcription factors (TF) were also induced by N luxuriant conditions. The most differentially expressed TF corresponded to an ortholog of *Arabidopsis Lateral Organ Boundary Domain* (*LBD38*). This result corroborates other studies showing that this particular TF and also other members of the family (*AtLBD37* and AtLBD39) were induced by nitrate in *Arabidopsis* [60] and by high N fertilization in poplar [10,13]. *AtLBD38 and also AtLBD37 and AtLBD39* were described to act as negative regulators of anthocyanin biosynthesis in *A. thaliana*, showing that these TFs could directly participate on phenylpropanoid transcriptional regulation [60].

### The high rates of lignification under N limiting fertilization are likely maintained by the flux of recycled NH_4_^+^

**Figure 7:**
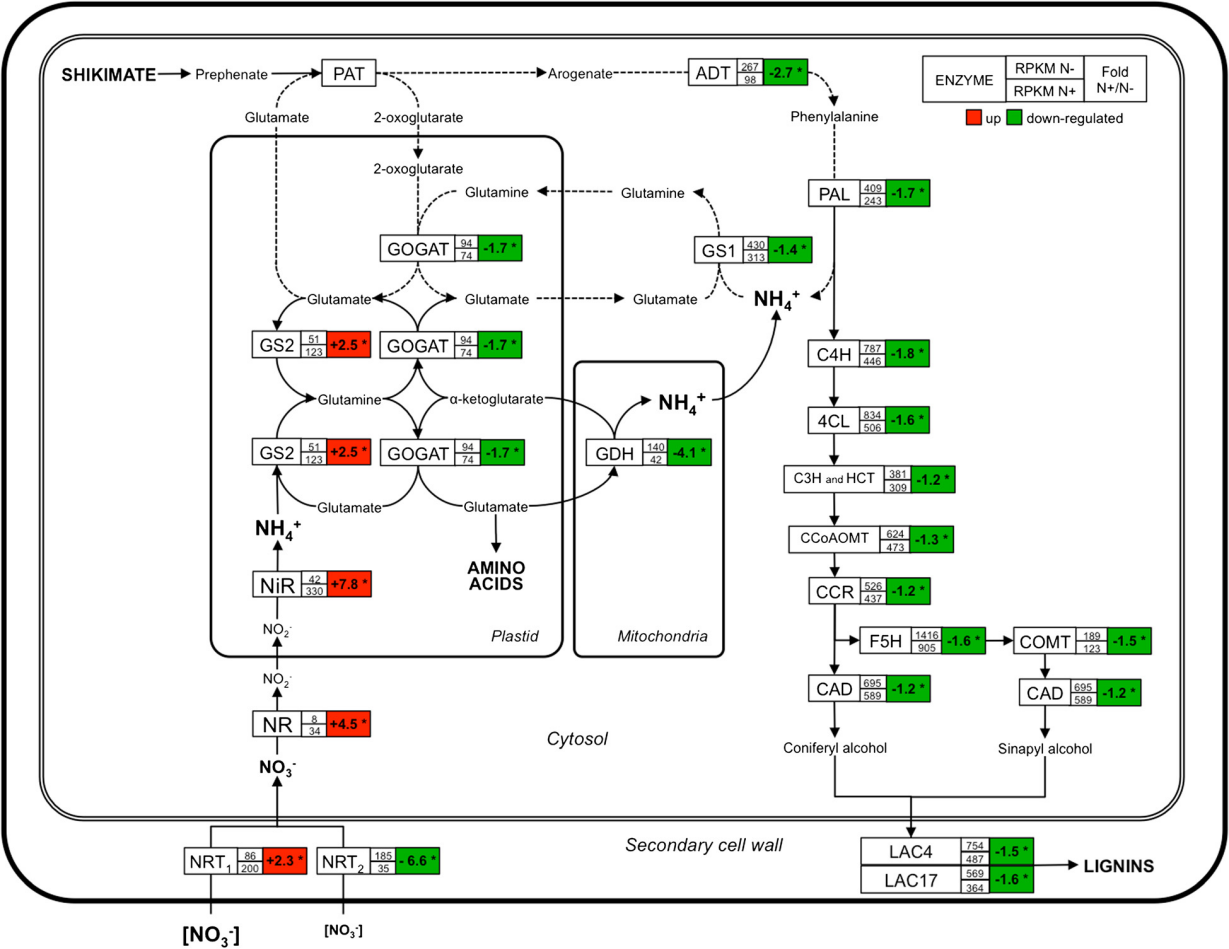
**Nitrogen and lignin metabolisms and expression profile from genes-related under contrasting N fertilization treatments.** Nitrate (NO_3_^−^) uptake is carried out by nitrate transporters (NRTs) and converted to nitrite (NO_2_^−^) by the nitrate reductase (NR) and in the plastid reduced to ammonium (NH_4_^+^). The NH_4_^+^ is incorporated by glutamine synthethase/glutamate synthase (GS2/GOGAT) into the amino acids. Cells in N deficiency and undergoing active lignification use an efficient N recycling mechanism where the NH_4_^+^ liberate by phenylalanine-ammonia lyase (PAL) and/or glutamate dehydrogenase (GDH) deamination by glutamate are incorporate by a cytosolic glutamine synthethase (GS1) and recycle into phenylalanine *via* Arogenate. Under N deficiency, high rates of lignification are maintained by the flux of N recycled. Expression profile by pairwise comparison between limiting (N-) and both luxuriant (N + and NO3) treatments; * = *P* < 0.01.

To get a comprehensive picture that could explain how lignification observed in N- plants is maintained and even enhanced, we schematized the nitrogen and the phenylpropanoid/lignin pathways in an integrated metabolism (Figure 7). We reported the expression ratios of all the genes involved in these metabolisms, even those genes that presented a fold change lower than ±1.5 cut-off but satisfied the *P*-value ≤0.01 (Additional file 2: Table S1).

In plants, nitrogen is assimilated from inorganic forms, NO_3_^−^ and NH_4_^+^, and incorporated into amino acids. NO_3_^−^ is up taken by nitrate transporters (NRTs) and converted to nitrite (NO_2_^−^) by the *Nitrate Reductase* (*NR*) and reduced to NH_4_^+^ by a plastidial *Nitrite Reductase (NiR).* As shown by the RNA seq data, the expression of all these genes involved in nitrogen metabolism exhibited the same trend, *i.e*. induced in the presence of high N fertilization. The only exception was *NRT2.5,* a high-affinity nitrate transporter that was up-regulated in limiting N supply.

In contrast to the plastidial GS2, two other *Glutamine synthetase 1* (*GS1*), one *Glutamate synthase* (*GOGAT*) and one *Glutamate dehydrogenase* (*GDH*) exhibited a pattern similar to *NRT2.5*. The GDH is potentially involved on glutamate deamination, whereas the cytosolic isoform of Glutamine synthetase (GS1) is involved in nitrogen assimilation and recycling [61]. The capacity of *GS1* to re-assimilate N from proteolysis and even from L-phenylalanine deamination has been described [61]. *GS1* has also been shown to co-express with *PAL* in conifer xylem cells [42,62,63]. The *PAL* genes, as well as the genes of the common phenylpropanoid metabolism and the lignin branch pathways, were induced in N limiting conditions.

These results suggest that an efficient N recycling mechanism, similar to that proposed by Canton et al. [42], in cells undergoing active lignification in trees, is induced in N limiting conditions and contributes to maintain a high rate of lignin biosynthesis. In this mechanism, the NH_4_^+^ liberated by *PAL* activity is incorporated by a cytosolic *glutamine synthethase* (*GS1*)/*GOGAT* and recycled into L-phenylalanine *via* Arogenate [42]. Recently, the *Prephenate aminotransferase* (PAT) gene was identified in *Arabidopsis* (At2g22250) [64,65]. Although *PAT* was not differently expressed between the contrasting N treatments, the increased transcript abundance of the 5 genes encoding *Arogenate dehydratases* in N limiting conditions further strengthens this hypothesis (Figure 7).

## Conclusions

We have shown here that N supply impacts SCW composition and structure, illustrating the high phenotypic plasticity of the SCW in response to nutrient availability. Among the differentially expressed genes between the two most contrasting treatments, limiting and luxuriant nitrogen conditions, those related to the phenylpropanoid and lignin biosynthesis were down-regulated in response to luxuriant nitrogen fertilization, in agreement with the phenotypic modifications observed. We were also able to propose that, in *Eucalyptus* under N deprivation, a mechanism involving the recycling of ammonium by arogenate is up-regulated in order to maintain the flux of nitrogen into lignin biosynthesis. Finally, we have shown that nitrogen fertilization, which is a common field management practice in commercial *Eucalyptus* forests, can be used not only to enhance biomass production but potentially used to modulate wood quality. This is particularly important for instance when lignocellulosic biomass is used for second-generation bioethanol production since lignin hinders cellulose accessibility during the saccharification step. Our findings thereby offers exiting possibilities to improve lignocellulosic biomass quantity and quality in *Eucalyptus*, making it more suitable for industrial end-uses by the means of field management practices such as N fertilization.

## Methods

### Plant material and nitrogen treatments

Three month-old rooted cuttings of a commercial *Eucalyptus urophylla × E. grandis* hybrid (clone IPB2-H15, kindly provided by International Paper do Brasil) were grown in greenhouse conditions (under natural light), in pots (7 liters) containing vermiculite. Three hundred trees exhibiting homogeneous size (20 cm) and developmental stage were selected and 75 plants were randomly attributed to each of the four nitrogen treatments.

The young trees were fertilized daily during 30 days with complete nutrient solution [66] where the level of N was adjusted with NH_4_NO_3_ to final concentrations of 7.5 mM for limiting N (N^−^), 15 mM for regular N (N) and 30 mM for luxuriant N (N+). In order to evaluate the potential impact of NO_3_ on monolignols biosynthesis, which has been described in Fritz et al. [67], a second luxuriant N treatment (NO_3_) was added where the final concentration of 30 mM of N was adjusted with KNO_3_ and Ca(NO_3_)_2_.4H_2_O.

The sampling was performed at the first hours of the day, and each treatment where randomly sampled in order to avoid possible artifacts associated to diurnal effects on gene expression. For RNA extraction, the stems of the *Eucalyptus* plants were harvested and after removing the bark, the xylem tissues were immediately frozen in liquid nitrogen and stored at −80°C. For each N treatment, 60 eucalypts plants were sampled and pooled into three biological replicates (n = 20). One pooled sample per treatment were used to individual RNA library sequencing, while three individual pooled samples (biological replicates) per N treatment were used for the qRT-PCR validation (Additional file 5: Table S4).

For the remaining analyses, 3 pools of 5 stems for each N treatment were harvested. Short stems segments (20 mm) were sampled at 5 cm of the basis of the stem of 5 plants per N treatment, fixed at FAA 50 buffer (Formaldehyde, Acetic Acid, Ethanol 50% - 1:1:18 v/v) for 48 h and transferred to ethanol 70% until the histological analyses.

For chemical analysis, the stems had their bark removed and the differentiating xylem tissue was scrapped in order to enrich the samples with cells produced during the treatment. All the samples were frozen in liquid nitrogen, lyophilized and ground to a powder in a ball mill (Tecnal Ltda) before analyses.

### Histological analysis

Stem transverse sections (80 μm thick) were obtained with a Sapphire knife on an automatic vibrating blade microtome (Leica VT 1000S). Sections were stained with the Weisner reagent (phloroglucinol-HCl) and immediately observed under bright-field microscopy (DM IRBE, Leica) coupled with a CCD camera (DFC 300 FX, Leica).

For scanning electron microscopy (SEM), samples were dehydrated in an ethanol series and submitted to a critical-point dry with CO_2_ as a transitional fluid. The dried tissues were coated with gold-palladium (20 nm) in a Jeol JFC1100 and analyzed using a scanning electron microscope (Hitachi S450) at 15KV.

### Chemical analysis

The lignin content (both total and Klason) was predicted using NIR-PLSR models. NIR spectra were collected in a Bruker-MPA spectrometer equipped with an integration sphere. The spectra were collected with a resolution of 8 cm^−1^ over the wave number range (12500 – 4000 cm^−1^) and for each spectrum 100 scans were taken. Analytical pyrolysis was performed using a CDS Pyroprobe 1000 with a coil filament probe connected to a GC (Agilent 6890) with flame ionization via a heated interface (270°C). The pyrolysis was carried out at 600°C for 5 s, using 75 μg of the extractive-free milled samples. Details of the conditions and quantification procedures have been published elsewhere [68-71].

### RNA extraction

The total RNA was extracted according to Le Provost et al. [72]. RNA quantity and quality was checked with a Nanodrop ND-1000 Spectrophotometer (Thermo) and 2100 Bioanalyzer (Agilent).

### Illumina sequencing

The mRNA-seq libraries were prepared at the High Throughput Sequencing Facility at Center of Genome, University of North Carolina, USA, using 10 μg of total RNA according to the Illumina’s protocol instructions, following a room temperature gel extraction step to avoid under-representation of AT-rich sequences [73]. The quality and quantity of each library were verified by Bioanalyzer Chip DNA 1000 series II (Agilent). For each library, one lane of an Illumina Genome Analyser IIx was used to produce the 36 bp single-end sequences. The complete dataset of RNA-seq reads has been deposited in SRA under accession numbers SRR1561174, SRR1561176, SRR1561161 and SRR1561153.

### Eucalyptus data set construction

The data set used in the analysis was constructed based on two public eucalyptus ESTs (Expressed Sequence Tags) database and a *de novo* assembly from our RNA sequencing reads. The Eucatoul (http://www.polebio.lrsv.ups-tlse.fr/eucatoul) contents 17,079 unigenes (7,921 contigs and 9,158 singlets) based on wood-related [74] and cold-tolerant [75] libraries. The Eucspresso (http://eucgenie.org) consists in an expressed gene catalog from a commercial clone of the hybrid *Eucalyptus grandis* × *E. urophylla* containing 18,894 contigs greater than 200 bp (18,606 with significant similarity with the *E. grandis* genome) [18].

To perform the *de novo* assembly, the reads produced by Illumina sequencing from all the four libraries were aligned against the filtered public data set using SOAP2 aligner [76]. The reads that did not map were then assembled using the Trinity program [77] configured to allow contigs with at least 200 bp and turning on the parameter “--run_butterfly”.

An automatic annotation of all contigs was performed using the Autofact program [78]; capable to resume the annotation based on sequence similarity searches in several databases. For this, the BLASTx [79] (e-value cutoff of 1e-5) was used to align the contigs against some public databases, including: non-redundant (NR) database of *NCBI*, Uniref90 and Uniref100 – databases containing clustered sets of proteins from Uniprot [80], Pfam – database of proteins families [81], KEGG – database of metabolic pathways [82] and TAIR (version 10) – database of *Arabidopsis* proteins. Functional annotation (GO) was performed using BLAST2GO [83] with default parameters.

### Reads alignment and gene expression statistical analysis

The Illumina reads were aligned against the data set (public and *de novo*) using the program SOAP2 [76], configured to allow up to two mismatches (SNPs can generate mismatches in the alignment, especially in our cases where the sequences originate from a hybrid), discard sequences with “N”s and return all optimal alignments.

The expression level for each contig was estimated using the “reads per kilobase of exon per million fragments mapped” (RPKM) value [84]. Pairwise comparisons were performed between the RPKM values from each library, using a T test at 99% of confidence rate (cut off of 0.01). Expression data of the differentially expressed genes (DEG) were log_2_ transformed and further standardized using the EXPANDER 6 software [85], which recalculates all gene RPKM values by giving a mean of 0 and a variance of 1 for each gene and taking into account the four treatments. This function is important to better distinguish gene expression differences between treatments. Standardized log2 RPKM of the DEG for each of the four N treatments were subjected to principal component analysis (PCA) enabling a graphical representation of the correlation between variables (DEG in each treatment). Functional annotation (GO) was performed using BLAST2GO [83] and all TAIR-annotated contigs were used in FunCat analysis [35].

### Data validation by RT-qPCR

RNA from each of the three biological replicates corresponding N + and N- *Eucalyptus* xylem were DNase treated and subjected to cDNA synthesis [33]. PCR primers are present in Additional file 5: Table S4. The high-throughput microfluidic qPCR assays were performed at the Genotoul Platform (http://genomique.genotoul.fr/) using the BioMark® 96:96 Dynamic Array (Fluidigm Corporation, San Francisco, CA, USA), as described in Cassan-Wang et al. [33]. The efficiency of each pair of primers was determined by plotting the Ct values obtained for serial dilutions of a mixture of all cDNAs and the equation Efficiency = 10^(−1/slope)^ − 1. The relative transcript abundance for each gene was calculated as described by Pfaffl [86], [(*E*_*target*_)^*ΔCt target* (*control* − *sample*)^/(*E*_*reference*_)^*ΔCt reference* (*control* − *sample*)^].The geometric mean of the ΔCts of five validated housekeeping genes [*SAND* (Eucgr.B02502), *PP2A1* (Eucgr.B03386), *EF1α* (Eucgr.B02473), *IDH* (Eucgr.F02901) and *PP2A3* (Eucgr.B03031)] was used as the reference [33]. Transcript abundance was standardized using the geometric mean of the corresponding N + and N- replicates as a control.

### Supporting data

The data set(s) supporting the results of this article is(are) included within the article (and its additional file(s)). The complete dataset of RNA-seq reads has been deposited in SRA under accession numbers SRR1561174, SRR1561176, SRR1561161 and SRR1561153.

## Additional files

Additional file 1: Figure S1. Eucalyptus data set construction and *de novo* assembly of xylem RNA reads. After Illumina-Solexa sequencing, more than 123 million xylem single-end reads (about 4.50 Gbp) of 36 bp length were obtained of which 116,360,230 (about 4.20 Gbp) remained for further analysis after filtering to exclude ribosomal and low quality reads. In order to map the produced reads of our RNA sequencing, we constructed a eucalyptus dataset based on two public eucalyptus ESTs databases [18,74,75]. The overlap between these two public databases was filtered resulting in 21,582 sequences. The Illumina-Solexa xylem reads were then aligned against the filtered public databases resulting in the successful mapping of 92,317,189 reads (79.34% of the total number of reads). Subsequently, a *de novo* assembly was performed with the remaining 24,043,041 unmapped reads, which produced 15,199 contigs greater than 200 bp. The final data set consisted of 36,781 unigenes. Additional file 2: Table S1 RPKM values for all trancripted genes. Additional file 3: Figure S2 Fold change distribution of all expressed genes. Additional file 4: Table S2 Differentially Expressed genes (Limiting vs Luxuriant treatments). Additional file 5: Table S4 RT-qPCR validation. Additional file 6: Table S3 Selected genes induced by nitrogen deprivation and/or repressed by nitrogen luxuriant supply; and repressed by nitrogen deprivation and/or induced by nitrogen luxuriant fertilization. (Level of expression in xylem; L-low, I-intermediate, H-high). Additional file 7: Figure S3 Top ten most represented Gene Ontology categories under “Biological Process” of genes induced by N deprivation and repressed by N luxuriant fertilization **(A and B)** and genes repressed by N deprivation and induced by N luxuriant fertilization **(C and D)**. The analyses were performed at level 3 **(A and C)** and level 4 **(B and D)**. Additional file 8: Table S5 Expression values of cellulose and hemicellulose/xylan transcripted genes. Genes that were considered differentially expressed (fold-change ≥ ±1.5 and P-value ≤ 0.01) are present in bold, genes that satisfied just the P-value cut-off (P-value ≤ 0.01) in regular font style.

## Abbreviations

4CL: 4-Coumarate:CoA ligase
ADT: Arogenate dehydratase
C3H: p-coumarate 3-hydroxylase
C4H: Cinnamate-4-hydroxylase
CAD: Cinnamyl alcohol dehydrogenase
CCoAOMT: Caffeoyl-CoA O-methyltransferase
CCR: Hydroxycinnamoyl-CoA reductase
COMT: Caffeate/5-hydroxyferulate O-methyltransferase
DEG: Differentially expressed genes
F5H: Ferulate 5-hydroxylase
FDH: Formate Dehydrogenase
GOGAT: Glutamate synthase
GS: Glutamine synthetase
HB: Hemoglobin
HCA: Hierarchical clustering analysis
HSP: Heat shock proteins
LBD: Lateral organ boundary domain
N: Nitrogen
NiR: Nitrite reductase
NIR: Near-infrared
NO: Nitric oxide
NR: Nitrate reductase
NRTs: Nitrate transporters
PAL: Phenylalanine ammonia lyase
PCA: Principal component analysis
qRT- PCR: Quantitative reverse transcription-PCR
RPKM: Reads per kilobase of exons per million of fragments mapped
SCW: Secondary cell wall
SEM: Scanning electron microscopy
S/G: Syringyl/guaiacyl ratio
SHMT: Serine Hydroxymethyltransferase 4
TF: Transcription factor
UPM1: Uroporphyrin III methyltransferase
