# Supplementary figures and images for: Contrasting nitrogen fertilization treatments impact xylem gene expression and secondary cell wall lignification in Eucalyptus
Source: BMC Plant Biol. 2014 Sep 28;14:256. doi: 10.1186/s12870-014-0256-9 (PMC4189757; doi:10.1186/s12870-014-0256-9)

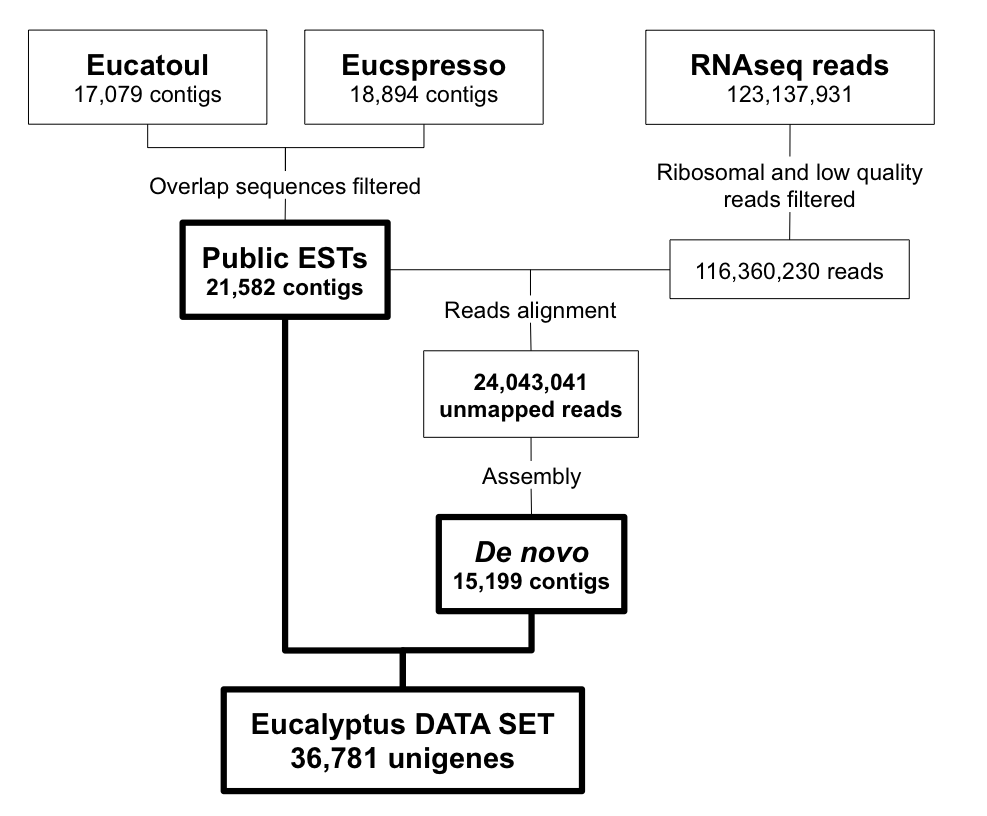

Supplement: Additional file 1: Figure S1. — Eucalyptus data set construction and de novo assembly of xylem RNA reads. After Illumina-Solexa sequencing, more than 123 million xylem single-end reads (about 4.50 Gbp) of 36 bp length were obtained of which 116,360,230 (about 4.20 Gbp) remained for further analysis after filtering to exclude ribosomal and low quality reads. In order to map the produced reads of our RNA sequencing, we constructed a eucalyptus dataset based on two public eucalyptus ESTs databases [18,74,75]. The overlap between these two public databases was filtered resulting in 21,582 sequences. The Illumina-Solexa xylem reads were then aligned against the filtered public databases resulting in the successful mapping of 92,317,189 reads (79.34% of the total number of reads). Subsequently, a de novo assembly was performed with the remaining 24,043,041 unmapped reads, which produced 15,199 contigs greater than 200 bp. The final data set consisted of 36,781 unigenes. [file 12870_2014_256_MOESM1_ESM.png]

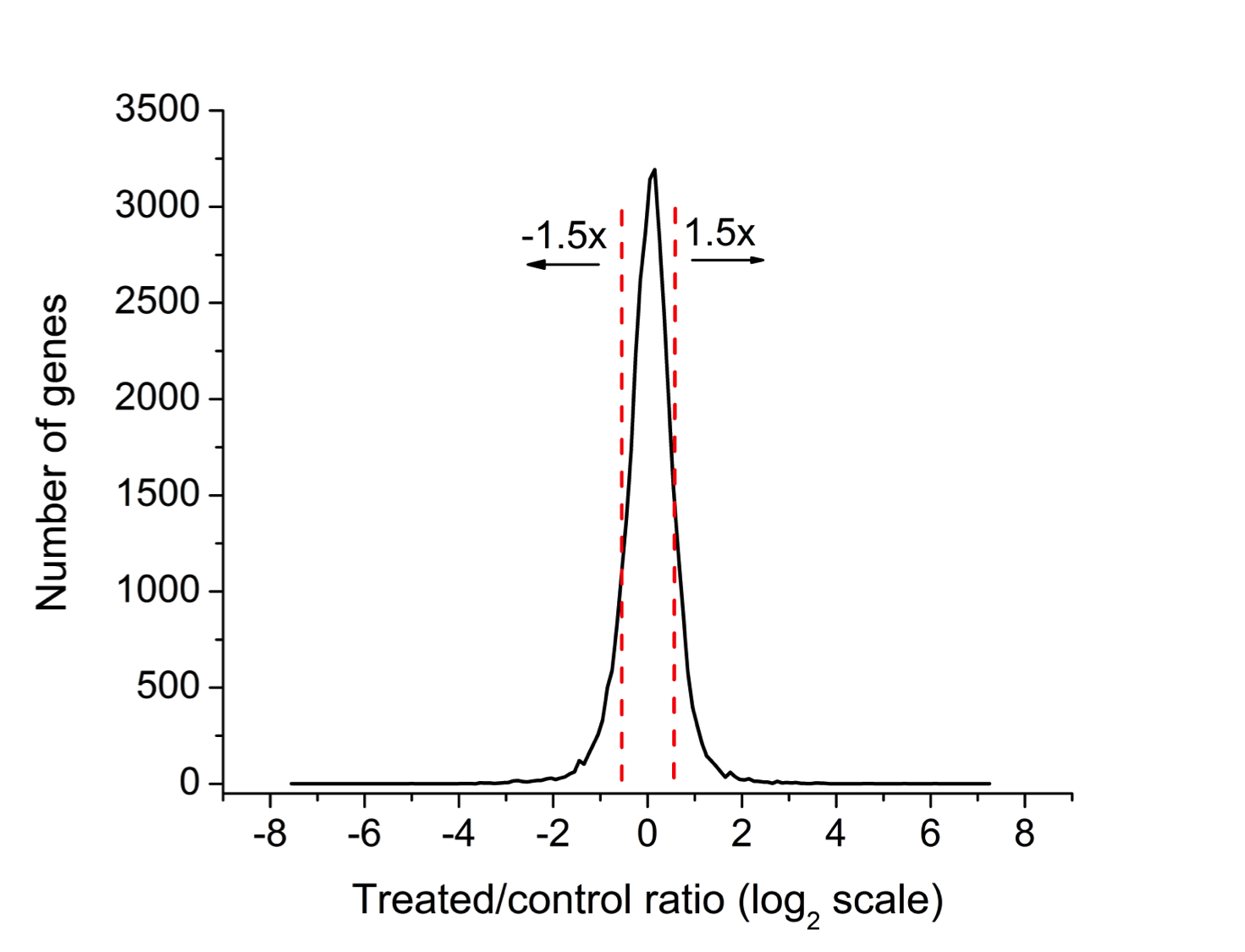

Supplement: Additional file 3: Figure S2 — Fold change distribution of all expressed genes. [file 12870_2014_256_MOESM3_ESM.png]

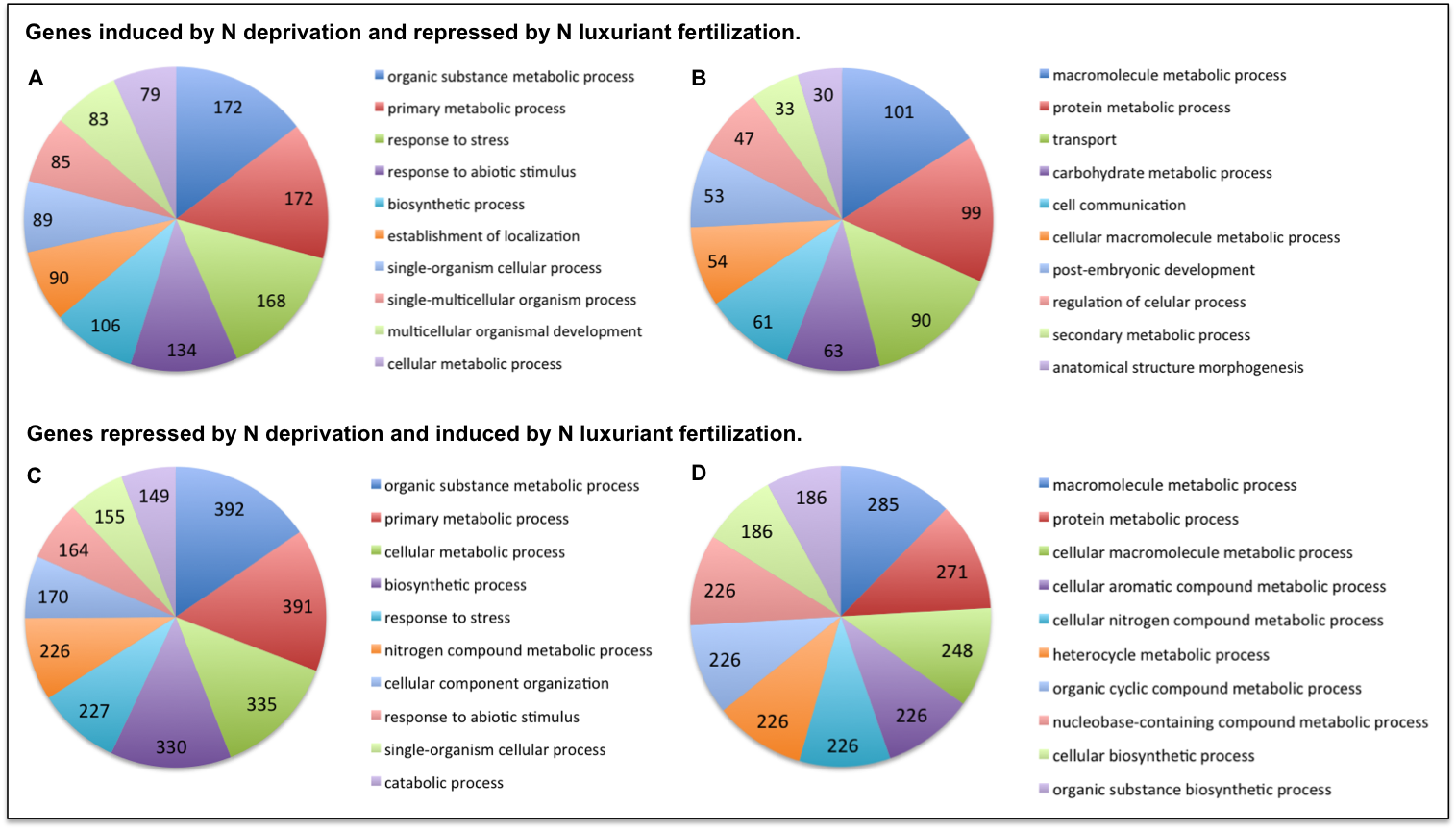

Supplement: Additional file 7: Figure S3 — Top ten most represented Gene Ontology categories under “Biological Process” of genes induced by N deprivation and repressed by N luxuriant fertilization (A and B) and genes repressed by N deprivation and induced by N luxuriant fertilization (C and D). The analyses were performed at level 3 (A and C) and level 4 (B and D). [file 12870_2014_256_MOESM7_ESM.png]
